# Efficacy and safety of drug-coated balloon angioplasty in proximal LAD stenosis with distal myocardial bridge: a 12-month comparative study with drug-eluting stents

**DOI:** 10.3389/fcvm.2025.1648215

**Published:** 2025-07-23

**Authors:** Xi Wu, Mingxing Wu, Haobo Huang, Zhe Liu, He Huang, Lei Wang

**Affiliations:** Department of Cardiology, Xiangtan Central Hospital (The Affiliated Hospital of Hunan University), Xiangtan, Hunan, China

**Keywords:** drug-coated balloon, drug-eluting stent, myocardial bridge, percutaneous coronary intervention, left anterior descending

## Abstract

**Introduction:**

Myocardial bridge (MB), particularly involving the distal left anterior descending artery (LAD), presents unique challenges in percutaneous coronary intervention (PCI) when coexisting with proximal LAD stenosis. Drug-coated balloon (DCB) represents a “leave nothing behind” strategy that may offer advantages over drug-eluting stents (DES) in these anatomically complex lesions.

**Methods:**

In this retrospective single-center study, 231 patients with proximal LAD stenosis and distal MB underwent PCI using either DCB (*n* = 83) or DES (*n* = 148). Intravascular ultrasound (IVUS)-guided lesion preparation and quantitative coronary angiography (QCA) were used. Clinical and angiographic outcomes were assessed at 12-month follow-up.

**Results:**

Baseline characteristics were comparable between groups. DES achieved greater acute lumen gain (1.94 ± 0.26 mm vs. 1.58 ± 0.36 mm; *p* < 0.001), while DCB resulted in significantly lower late lumen loss (−0.04 ± 0.04 mm vs. 0.18 ± 0.05 mm; *p *< 0.001). The incidence of major adverse cardiovascular events (MACE) was lower in the DCB group (9.6% vs. 21.6%; *p* = 0.033), as was target lesion revascularization (7.2% vs. 18.2%; *p* = 0.035).

**Conclusion:**

DCB angioplasty is a safe and effective alternative to DES in proximal LAD lesions complicated by distal MB. Despite reduced acute lumen gain, DCBs yielded comparable angiographic results, reduced restenosis and MACE, and enabled shorter dual antiplatelet therapy duration.

## Introduction

Coronary arteries are usually located on the epicardial surface of the heart. However, in certain individuals, congenital anomalies can result in segments of these arteries penetrating the myocardial tissue. This intramyocardial segment, enveloped by myocardial fibers, is referred to as a myocardial bridge (MB) (1). During systolic contraction, the bridged portion—also termed the mural coronary artery—may undergo compression, potentially leading to transient luminal narrowing or even complete occlusion (2). The presence of MB was first documented by Geiringer in 1951 during postmortem examinations (3). Although traditionally regarded as a benign anatomical variant, increasing evidence from advanced imaging technologies suggests that MB is implicated in various cardiovascular pathologies, such as acute coronary syndrome (ACS) (4), coronary vasospasm (5), ventricular arrhythmias (6), premature atherosclerosis, and even sudden cardiac death (7). Prevalence rates of MB show considerable variability depending on the detection method: approximately 6% with conventional coronary angiography (CAG), 22% using computed tomography angiography (CTA), and as high as 42% in autopsy studies (8). The repetitive mechanical compression imposed by MB during systole alters local shear stress dynamics, facilitating the development of atherosclerotic plaques proximal to the bridged region. Notably, up to 86% of these lesions occur before the MB, and approximately 67%–98% of MBs are located in the mid-segment of the left anterior descending (LAD) artery (1, 9).

Of particular concern is the proximal LAD, given its role in perfusing critical myocardial territories, including the anterior wall, interventricular septum, and apex (10). Any stenosis in this region poses a substantial threat to myocardial function and long-term prognosis (10). When proximal LAD disease coexists with a distal MB, therapeutic decision-making becomes more complex, as interventions may disrupt downstream hemodynamics and MB-related flow characteristics (2, 11, 12). At present, revascularization strategies targeting MB-associated lesions are not standardized (1). While drug-eluting stents (DES) are frequently employed, their use in MB anatomy is often associated with suboptimal outcomes. Clinical data indicate an increased incidence of major adverse cardiovascular events (MACEs) (13), in-stent restenosis (ISR) (14), and stent fractures in patients with MB compared to those without. Additionally, prolonged dual antiplatelet therapy (DAPT) required post-stenting raises bleeding risks, particularly among elderly or comorbid populations (10).

Recently, drug-coated balloons (DCB) have emerged as a promising non-stent-based modality, aligned with the “leave nothing behind” treatment paradigm (15). These semi-compliant devices deliver lipophilic antiproliferative drugs directly to the vessel wall, avoiding the long-term implications of permanent metallic implants (16). DCBs have demonstrated efficacy in treating small vessel disease (17, 18), ISR (19), and *de novo* lesions (20). In the context of MB, DCBs offer theoretical benefits such as preserving physiological vessel compliance, minimizing mechanical disruption, and potentially improving procedural safety in anatomically complex regions.

Nevertheless, clinical evidence supporting the use of DCBs in lesions located proximal to MB remains scarce—particularly in the setting of proximal LAD stenosis complicated by distal MB. This unique anatomical configuration combines static luminal obstruction with dynamic systolic compression, presenting a therapeutic challenge (21). The absence of robust comparative studies has left clinicians without clear guidance for optimal revascularization strategies in such scenarios (16). Therefore, the present study seeks to compare the efficacy and safety of DCB vs. DES in managing proximal LAD lesions with concurrent distal MB. By evaluating clinical endpoints, hemodynamic performance, and complication rates, this investigation aims to provide evidence-based recommendations for treating this complex subset of coronary artery disease (CAD).

## Materials and methods

### Study participants

**Figure 1:**
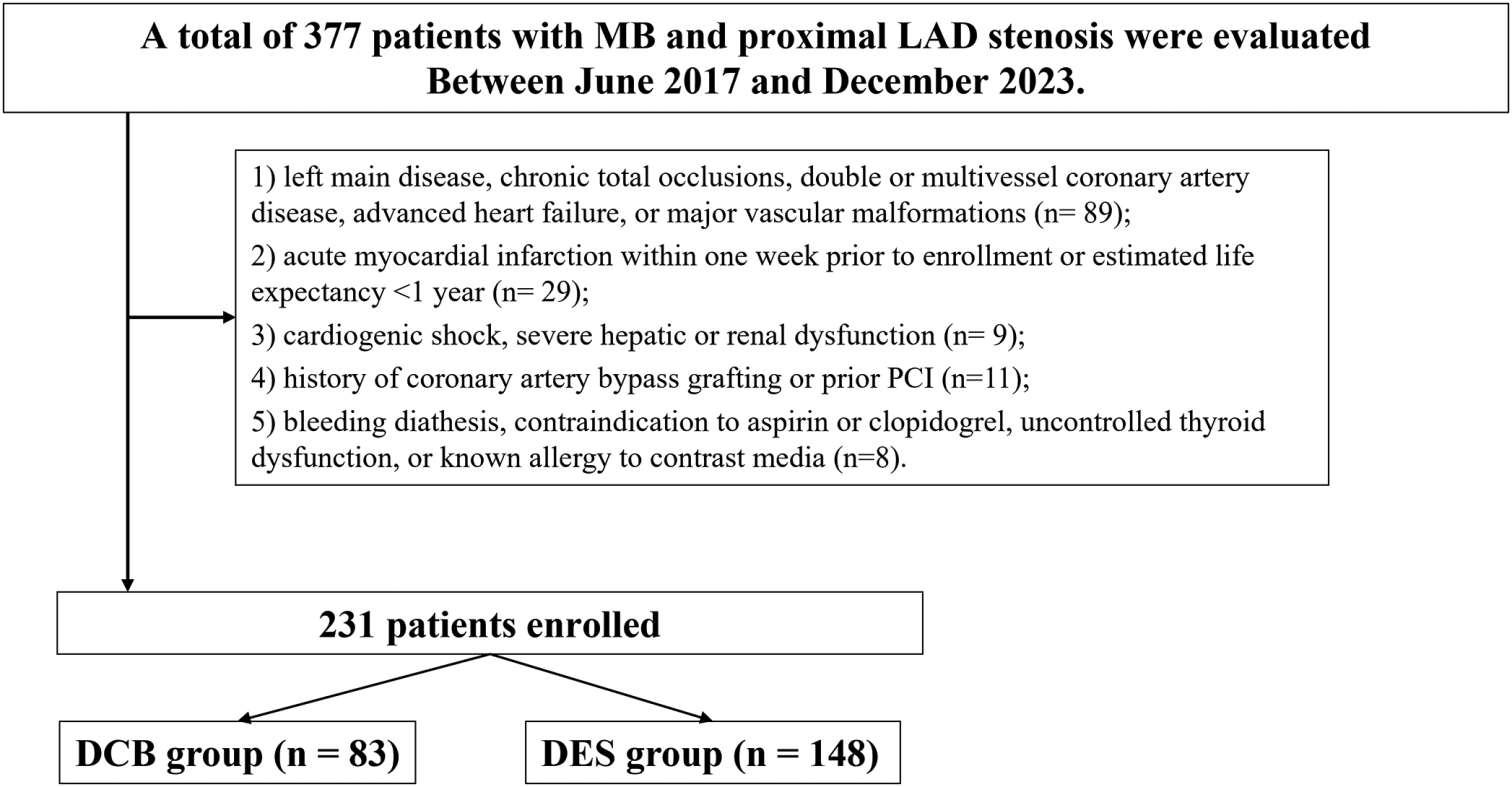
Study flowchart. MB, myocardial bridge; PCI, percutaneous coronary intervention; DES, drug-eluting stents; DCB, drug-coated balloons; LAD, left anterior descending.

This research was a retrospective, single-center observational study conducted within the Department of Cardiology at Xiangtan Central Hospital. From June 2017 to December 2023, patients hospitalized with ACS were evaluated via CAG to identify cases presenting with proximal LAD artery stenosis in conjunction with distal MB. Inclusion required a confirmed diagnosis consistent with the criteria set forth by the American College of Cardiology (ACC) and the American Heart Association (AHA) for unstable angina (UA), non–ST-segment elevation myocardial infarction (NSTEMI), ST-segment elevation myocardial infarction (STEMI), or stable CAD accompanied by objective indicators of myocardial ischemia and a scheduled elective percutaneous coronary intervention (PCI) (22). Eligibility criteria were as follows: (1) the presence of clinical manifestations indicative of myocardial ischemia, such as exertional chest pain or angina; (2) supporting evidence on electrocardiography (ECG) and/or echocardiography consistent with ischemic pathology; (3) angiographic confirmation of a single, significant proximal LAD lesion (≥70% stenosis) coexisting with a distal MB (1); and (4) characteristic findings of MB on CAG, defined by systolic luminal narrowing due to myocardial overlying fibers, resulting in partial or near-total compression during systole with subsequent diastolic lumen restoration—a phenomenon known as the “milking effect” (1). Patients were excluded if they met any of the following conditions: (1) presence of left main CAD, chronic total occlusion, multivessel involvement, advanced-stage heart failure, or congenital vascular anomalies; (2) acute MI within one week prior to enrollment or an estimated life expectancy of less than one year; (3) cardiogenic shock, severe hepatic or renal impairment (e.g., glomerular filtration rate <30 ml/min); (4) prior history of coronary artery bypass grafting (CABG) or PCI; or (5) known bleeding disorders, hypersensitivity to aspirin or clopidogrel, uncontrolled thyroid disease, or allergy to iodinated contrast agents (see Figure 1).

Demographic characteristics, clinical risk factors, laboratory data, angiographic and IVUS findings, procedural variables, and long-term follow-up outcomes were retrospectively extracted from the hospital's electronic health record and imaging archives. The study adhered to the ethical standards set by the Declaration of Helsinki (2013 revision) and received approval from the Ethics Committee of Xiangtan Central Hospital (Approval No. X2019452). Written informed consent was secured from all participants; where written consent could not be obtained, verbal consent was documented per institutional protocol.

### Periprocedural pharmacotherapy

All patients received standard DAPT, consisting of aspirin (100 mg/day) and clopidogrel (75 mg/day), for a minimum of 7 days prior to the procedure. A pre-procedural loading dose was administered 24 h before intervention, comprising aspirin 300 mg along with either clopidogrel 300 mg or ticagrelor 180 mg, selected based on clinical discretion. Post-intervention antiplatelet regimens were determined by the revascularization strategy employed. In the DCB group, patients continued on DAPT with aspirin (100 mg/day) and clopidogrel (75 mg/day) for 1 month, after which they transitioned to single antiplatelet therapy (SAPT) using either aspirin or clopidogrel at standard daily doses for a duration of up to 12 months. Conversely, patients treated with DES maintained conventional DAPT—aspirin (100 mg/day) plus clopidogrel (75 mg/day)—for the entire 12-month follow-up period. Additional pharmacological therapies, including statins, beta-blockers, angiotensin-converting enzyme inhibitors (ACEIs) or angiotensin receptor blockers (ARBs), and nitrates, were prescribed based on clinical indications and tailored to the individual's cardiovascular risk profile.

### Interventional procedures

All procedures were conducted in the catheterization laboratory at Xiangtan Central Hospital by experienced interventional cardiologists. The radial artery served as the primary access route for vascular intervention. Prior to treatment, all patients underwent CAG to delineate coronary anatomy. Anatomical segmentation was standardized as follows: the proximal segment of the LAD was defined as the portion from the LAD ostium to the origin of the first diagonal branch (D1) (1), while the distal MB was defined as a bridged LAD segment located beyond the second diagonal branch (D2), characterized by systolic compression with diastolic restoration on CAG (23). Intravascular ultrasound (IVUS) was used intraoperatively to further evaluate lesion characteristics, including plaque composition, stenosis severity, calcification, and the spatial relationship between the proximal stenotic lesion and the distal MB.

### DCB group

Patients in the DCB group were managed in accordance with the recommendations outlined in the Third Report of the International DCB Consensus Group (16). In this study, the DCB used was the SeQuent Please™ paclitaxel-coated balloon (B. Braun Melsungen AG, Germany), available in diameters of 2.0–4.0 mm and lengths of 10–30 mm. All balloons were inflated at nominal pressure (8–10 atm) and maintained for 30–60 s. For lesions with minimal plaque burden and hemodynamic stability, lesion preparation was performed using semi-compliant balloons. In cases presenting with a substantial thrombotic burden, manual aspiration thrombectomy was employed to reduce thrombus load. For lesions exhibiting fibrotic or severe calcific features, lesion modification was achieved using non-compliant balloons, cutting balloons, or scoring balloons, with the goal of optimizing plaque modification while limiting the risk of major dissection. Lesion preparation was deemed adequate if the following criteria were met: absence of dissection or only type A/B dissection per the National Heart, Lung, and Blood Institute (NHLBI) classification, restoration of TIMI grade 3 flow, and residual stenosis of 30% or less (16). After predilatation, a 5–10 min observation period was instituted to monitor for elastic recoil, during which intracoronary nitroglycerin was administered to evaluate vessel tone and ensure hemodynamic stability. To avoid geographic mismatch, the balloon-to-vessel diameter ratio was maintained within the range of 0.8–1.0, and balloon length was selected to extend at least 2–3 mm beyond both proximal and distal lesion margins, covering a total excess of ≥5 mm. DCBs were deployed at nominal pressure and maintained for an inflation duration of 30–60 s. Procedural success was defined as post-procedural TIMI 3 flow, residual stenosis ≤30%, and the absence of flow-limiting dissection. In instances of significant vessel injury—such as type C or higher dissection, persistent luminal compromise, residual stenosis >30%, or TIMI flow <3—a bailout implantation of DES was performed.

### DES group

In the DES group, patients underwent conventional PCI with stent implantation (24). The DES included XIENCE™ everolimus-eluting stents (Abbott Vascular, USA) and Resolute Integrity™ zotarolimus-eluting stents (Medtronic, USA), selected at the discretion of the operator. Stents were available in diameters of 2.25–4.0 mm and lengths ranging from 8 to 38 mm, with deployment at nominal to high pressure (12–18 atm) based on vessel size and lesion characteristics. Lesion preparation was guided by IVUS. Semi-compliant balloons were used for softer lesions, while non-compliant, cutting, or scoring balloons were applied in fibrotic or calcified segments to ensure optimal dilation. When thrombus was present, aspiration thrombectomy was performed. Stent size was selected based on vessel reference diameter and lesion length, with 2–3 mm margin beyond both ends of the lesion to ensure full coverage. Stents were deployed at nominal to high pressure, followed by post-dilatation using non-compliant balloons to improve stent apposition and expansion. Procedural success was defined as: TIMI grade 3 flow, <30% residual stenosis, good stent apposition on IVUS. DAPT was administered post-procedure per guideline recommendations, unless contraindicated.

The selection of DCB vs. DES was determined by the operating interventional cardiologist at the time of the procedure, based on a combination of anatomical and clinical factors. Specifically, DCB therapy was preferred in lesions with favorable morphology (e.g., shorter length, non-calcified plaques, no flow-limiting dissection after predilation), good vessel size match, and adequate lesion preparation without geographic mismatch. DES was selected in cases with complex anatomy (e.g., heavy calcification, diffuse disease), poor predilation results, or bailout indication (e.g., type C dissection or significant residual stenosis). Although institutional practice generally favored DCB in appropriate lesions, there was no formal randomization or pre-specified protocol governing device allocation. All decisions were made in accordance with current clinical guidelines and IVUS-based assessment.

### IVUS imaging and analysis

In all patients assigned to the PCI group, IVUS imaging was performed prior to stent deployment. In this study, IVUS was systematically performed in all enrolled patients (100%), regardless of treatment assignment. Specifically, all 83 patients in the DCB group and all 148 patients in the DES group underwent IVUS evaluation, with no cases excluded due to technical limitations or operator discretion. The application of IVUS followed a standardized institutional protocol and was used in all cases for lesion characterization, procedural planning, and quantitative analysis. Accordingly, there were no significant differences in IVUS usage frequency, image acquisition protocols, or analytic parameters between the two groups. This ensures methodological consistency and minimizes potential bias related to imaging heterogeneity. Following successful guidewire passage, an intracoronary bolus of nitroglycerin (100–200 μg) was administered to minimize vasomotor tone and enhance image clarity. Two IVUS catheter systems were utilized during the study period: from 2017 to 2019, the 40 MHz Atlantis SR (Boston Scientific, USA), and from 2019 to 2022, the OptiCross catheter (Boston Scientific, USA). Both systems were compatible with the iLab IVUS console and offered equivalent image quality and acquisition protocols. The IVUS catheter was advanced distal to the lesion of interest and withdrawn proximally under fluoroscopic visualization at a standardized pullback rate of 0.5–1.0 mm/s. All imaging sequences were digitally recorded for subsequent offline analysis. Quantitative assessment was conducted using QIvus® software (Medis, Leiden, the Netherlands) by two independent observers blinded to patient clinical information. In cases of interpretative disagreement, consensus was reached via adjudication by a third senior reviewer. To ensure methodological uniformity, identical acquisition protocols and analytic parameters were consistently applied across all cases. MB was defined by IVUS as a tunneled segment of an epicardial coronary artery exhibiting systolic compression and encasement within echolucent muscular tissue (23). For each case, the following parameters were analyzed: minimum lumen area (MLA) at the stenotic segment, plaque burden at the MLA site, maximum thickness of the overlying myocardial fibers, total MB length, and diastolic vessel restriction, calculated as (1—diastolic vessel area/interpolated reference area) (23). IVUS guidance was used for stent optimization, ensuring placement in vessel segments with plaque burden less than 50%. Stent expansion was assessed by calculating the ratio between the minimum stent area (MSA) and the average lumen area of adjacent reference segments (proximal and distal). Care was taken to avoid extending the stent into the bridged segment unless clinically necessary, such as in the event of significant proximal dissection. All IVUS-derived metrics were acquired during presumed end-diastole to standardize inter-patient comparisons. Final anatomical evaluations from both IVUS and angiography were independently reviewed by two experienced interventional cardiologists (X.W. and H.H.), both blinded to treatment assignment. The reproducibility of measurements was high, with inter- and intra-observer *κ* values of 0.89 and 0.92, respectively.

### Follow-up and outcome measures

All patients were followed for a duration of 12 months through outpatient clinic visits or structured telephone interviews to monitor the occurrence of MACEs and minor bleeding complications (25). At the 12-month follow-up, all participants underwent repeat CAG to assess the presence of ISR, neointimal hyperplasia, thrombus formation, and healing of any previously observed dissections. Quantitative coronary angiography (QCA) was employed to perform serial measurements—pre-procedural, immediately post-procedural, and at follow-up—of the following angiographic indices: minimum lumen diameter (MLD), acute lumen gain, and late lumen loss (LLL). MACEs were defined according to the standardized criteria established by the Academic Research Consortium (ARC) (26), and included events such as rehospitalization for recurrent angina, MI related to the target vessel, cardiac death, and clinically driven target lesion revascularization (TLR). Acute lumen gain was defined as the difference between the post-PCI MLD and the pre-PCI MLD, reflecting the immediate procedural efficacy (27). LLL was defined as the difference between the post-PCI MLD and the MLD measured during follow-up angiography, serving as an indicator of long-term vessel patency and neointimal proliferation (27).

### Statistical analysis

All statistical analyses were conducted using IBM SPSS Statistics, version 26.0 (IBM Corp., Armonk, NY, USA). The distribution of continuous variables was assessed using the Kolmogorov–Smirnov test to determine normality. Data following a normal distribution were expressed as mean ± standard deviation (SD) and compared between groups using independent-samples *t*-tests. For non-normally distributed data, results were presented as median with interquartile range (IQR) and analyzed using the Mann–Whitney *U* test. Categorical variables were summarized as frequencies and percentages, with group comparisons performed using either Pearson's chi-squared test or Fisher's exact test, depending on expected cell counts. Variables found to be statistically significant in univariate analysis were subsequently included in a multivariate Cox proportional hazards regression model employing a backward stepwise selection approach to identify independent predictors of MACE. In addition, to adjust for procedural imbalances, variables related to lesion preparation techniques (e.g., use of cutting or scoring balloons) were also included in the multivariate model. To minimize reverse causality bias, the Cox regression analysis involving LLL was restricted to patients who remained free from MACE up to the 12-month follow-up angiography, thereby ensuring that LLL values reflected pre-event measurements. Time-to-event analyses were carried out using Kaplan–Meier survival curves, and differences between groups were assessed via the log-rank test. A two-tailed *p*-value of less than 0.05 was considered indicative of statistical significance.

## Results

### Baseline characteristics

A total of 231 patients were included, comprising 83 in the DCB group and 148 in the DES group. The median age was comparable between groups (62.65 vs. 63.58 years, *p* = 0.129), as was the proportion of males (55.4% vs. 64.9%, *p* = 0.202). No statistically significant differences were observed in major cardiovascular risk factors, including hypertension (39.8% vs. 44.6%, *p* = 0.566), diabetes (37.3% vs. 31.1%, *p* = 0.409), or history of MI (26.5% vs. 24.3%, *p* = 0.834). Laboratory values and medication use were also comparable (Table 1).

**Table 1:** Baseline characteristics.

Variable	All (*n* = 231)	DCB group (*n* = 83)	DES group (*n* = 148)	*P* value
Age, years	63.32 (61.07, 64.99)	62.65 (61.13, 64.56)	63.58 (61.07, 65.15)	0.129
Male, *n*%	142 (61.5%)	46 (55.4%)	96 (64.9%)	0.202
Prior hypertension, *n*%	99 (42.9%)	33 (39.8%)	66 (44.6%)	0.566
Prior hyperlipidemia, *n*%	75 (32.5%)	29 (34.9%)	46 (31.1%)	0.649
Prior diabetes mellitus, *n*%	77 (33.3%)	31 (37.3%)	46 (31.1%)	0.409
Prior stroke, *n*%	13 (5.6%)	3 (3.6%)	10 (6.8%)	0.485
Smoking, *n*%	113 (48.9%)	41 (49.4%)	72 (48.6%)	1.000
Chronic kidney diseasea^a^, *n*%	13 (5.6%)	7 (8.4%)	6 (4.1%)	0.276
Peripheral artery disease, *n*%	22 (9.5%)	4 (4.8%)	18 (12.2%)	0.111
Prior myocardial infarction, *n*%	58 (25.1%)	22 (26.5%)	36 (24.3%)	0.834
Prior PCI, *n*%	32 (13.9%)	12 (14.5%)	20 (13.5%)	0.997
Laboratory biomarkers				
Platelet count, 10^9^/L	249.33 (240.34, 258.94)	247.71 (239.50, 257.45)	250.90 (241.21, 259.92)	0.162
TG, mmol/L	1.69 (0.94, 2.50)	1.49 (0.89, 2.36)	1.91 (0.97, 2.61)	0.075
TC, mmol/L	5.30 (5.18, 5.46)	5.29 (5.17, 5.46)	5.31 (5.18, 5.47)	0.814
HDL, mmol/L	1.26 (1.19, 1.32)	1.27 (1.18, 1.34)	1.25 (1.19, 1.31)	0.202
LDL, mmol/L	3.37 (3.22, 3.48)	3.39 (3.24, 3.48)	3.36 (3.22, 3.48)	0.613
Lp(a), mg/L	198.89 (187.72, 210.18)	197.78 (188.88, 207.33)	199.26 (187.00, 210.86)	0.571
AST, U/L	112.93 (105.65, 120.69)	113.40 (106.41, 123.10)	112.86 (105.10, 120.27)	0.366
ALT, U/L	49.52 (44.09, 54.25)	49.18 (42.84, 54.16)	49.68 (44.17, 54.27)	0.712
TBIL, umol/L	16.37 (15.37, 17.29)	16.41 (15.75, 17.34)	16.36 (15.05, 17.29)	0.248
Uric acid, umol/L	476.03 (455.49, 491.03)	475.38 (455.26, 487.47)	478.73 (458.02, 493.75)	0.143
Scr, µmol/L	89.20 (86.92, 91.54)	89.30 (87.18, 91.91)	88.83 (86.47, 91.19)	0.358
eGFR, ml/min per 1.732 m^2^	99.62 (95.73, 103.57)	99.30 (95.20, 103.80)	99.81 (95.92, 103.37)	0.734
Pharmacologic therapy				
DAPT, *n*%	231 (100%)	83 (100.0%)	148 (100.0%)	1.000
Statins, *n*%	213 (92.2%)	78 (94.0%)	135 (91.2%)	0.620
ACEI or ARB, *n*%	132 (57.1%)	46 (55.4%)	86 (58.1%)	0.796
Beta-blockers, *n*%	187 (81.0%)	69 (83.1%)	118 (79.7%)	0.647
Aldosterone antagonists, *n*%	30 (13.0%)	10 (12.0%)	20 (13.5%)	0.909
Nitrates, *n*%	7 (3.0%)	4 (4.8%)	3 (2.0%)	0.430
Calcium channel blockers, *n*%	23 (10.0%)	6 (7.2%)	17 (11.5%)	0.419

^a^ Estimated glomerular filtration rate <60 ml/min/1.73 m^2^ using the Modification of Diet in Renal Disease study equation.

Continuous variables were expressed as median (interquartile range). Categorical variables were expressed as number (percentage).

DES, drug-eluting stents; DCB, drug-coated balloons; PCI, percutaneous coronary intervention; DAPT, dual antiplatelet therapy; ACEI, angiotensin-converting enzyme inhibitor; ARB, angiotensin-receptor blocker; TG, triglycerides; TC, total cholesterol; HDL, high-density lipoprotein; LDL, low-density lipoprotein; Lp(a), lipoprotein(a); AST, aspartate aminotransferase; ALT, alanine aminotransferase; TBIL, total bilirubin; Scr, serum creatinine; eGFR, estimated glomerular filtration rate;.

### Angiographic and procedural findings

Lesion characteristics were largely balanced between groups, including lesion length (25.93 ± 3.52 mm vs. 26.34 ± 3.62 mm, *p* = 0.412) and proximal stenosis severity (78.58% vs. 78.72%, *p* = 0.877). However, significant differences were noted in device selection: non-compliant high-pressure balloons were more frequently used in the DES group (71.6% vs. 15.7%, *p* < 0.001), while cutting balloons (63.9% vs. 20.9%, *p* < 0.001) and scoring balloons (32.5% vs. 4.1%, *p* < 0.001) were more common in the DCB group. Dissection types A–B were more frequent in the DCB group (14.5% vs. 1.4%, *p* < 0.001). Post-procedure vessel diameters and stenosis percentages were similar across groups (e.g., minimum lumen diameter: 2.60 mm vs. 2.58 mm, *p* = 0.518) (Table 2).

**Table 2:** Angiographic and procedural findings.

Variable	All (*n* = 231)	DCB group (*n* = 83)	DES group (*n* = 148)	*P* value
Proximal stenosis severity, %	78.67 ± 6.77	78.58 ± 7.60	78.72 ± 6.26	0.877
Lesion length >20 mm, *n*%	60 (26.0%)	20 (24.1%)	40 (27.0%)	0.740
Lesion length, mm	26.19 ± 3.59	25.93 ± 3.52	26.34 ± 3.62	0.412
MB length, mm	11.31 ± 4.31	11.63 ± 4.41	11.13 ± 4.24	0.403
Systolic compression severity of the bridged segment, %	63.31 ± 7.22	62.82 ± 7.18	63.58 ± 7.24	0.449
Distance from proximal stenosis to the myocardial bridge, mm	14.40 ± 3.13	14.33 ± 2.88	14.45 ± 3.26	0.789
Calcification, *n*%	26 (11.3%)	8 (9.6%)	18 (12.2%)	0.714
Calcium length, mm	10.06 ± 0.55	10.11 ± 0.55	10.04 ± 0.54	0.334
Lesion bend, *n*%	32 (13.9%)	12 (14.5%)	20 (13.5%)	0.993
Non-compliant high-pressure balloon, *n*%	119 (51.5%)	13 (15.7%)	106 (71.6%)	<0.001
Cutting balloon, *n*%	84 (36.4%)	53 (63.9%)	31 (20.9%)	<0.001
Scoring balloon, *n*%	33 (14.3%)	27 (32.5%)	6 (4.1%)	<0.001
Dissection, *n*%				
type A–B	4 (1.7%)	12 (14.5%)	2 (1.4%)	<0.001
type C–F	1 (0.4%)	1 (1.2%)	0 (0.0%)	0.359
Elastic recoil of the vessel, *n*%	1 (0.4%)	1 (1.2%)	0 (0.0%)	0.359
Post-PCI in-segment^a^				
Reference vessel diameter, mm	3.22 ± 0.52	3.13 ± 0.48	3.27 ± 0.54	0.055
Minimum lumen diameter, mm	2.59 ± 0.20	2.60 ± 0.21	2.58 ± 0.19	0.518
Diameter stenosis, %	20.93 ± 3.32	20.68 ± 3.65	21.08 ± 3.12	0.383
Post-PCI distal vessel				
Reference vessel diameter, mm	2.00 ± 0.97	2.01 ± 0.86	1.99 ± 1.02	0.872
Minimum lumen diameter, mm	1.14 ± 0.30	1.15 ± 0.29	1.14 ± 0.31	0.773
Diameter stenosis, %	28.37 ± 3.37	28.58 ± 3.55	28.25 ± 3.27	0.483
Procedural findings				
Total stent length, mm	28.89 ± 5.82	29.22 ± 6.19	28.70 ± 5.59	0.518
Stent extension into MB, *n*%	–	–	43 (29.0%)	–
Maximum device diameter, mm	3.07 ± 2.36	3.33 ± 2.19	2.92 ± 2.45	0.214
Maximum balloon inflation pressure, atm	18.59 ± 3.08	18.76 ± 2.90	18.50 ± 3.17	0.536
Procedure time, min	45.97 ± 6.70	45.83 ± 6.18	46.04 ± 6.98	0.821
Radiation exposure dose, Gy	1.95 ± 0.31	1.97 ± 0.35	1.94 ± 0.28	0.495
Contrast media volume, ml	268.48 ± 14.37	266.89 ± 15.00	269.37 ± 13.93	0.211

^a^ In-segment includes stent and 5 mm proximal and distal reference from each stent edge.

Continuous variables were expressed as mean ± SD. Categorical variables were expressed as number (percentage).

DES, drug-eluting stents; DCB, drug-coated balloons; PCI, percutaneous coronary intervention; MB, myocardial bridge.

### Intravascular ultrasound findings

IVUS analysis showed no significant differences in lesion length (29.14 ± 5.51 mm vs. 28.67 ± 4.98 mm, *p* = 0.508), plaque burden (84.16% vs. 84.22%, *p* = 0.932), or reference lumen area. However, the DCB group had a higher incidence of dissection (16.9% vs. 2.0%, *p* < 0.001), while the DES group achieved a greater acute lumen gain (1.94 ± 0.26 mm vs. 1.58 ± 0.36 mm, *p* < 0.001). Post-procedure MSA (5.57 vs. 5.35 mm^2^, *p* = 0.581) and stent expansion (70.28% vs. 70.33%, *p* = 0.915) were comparable (Table 3).

**Table 3:** Intravascular ultrasound findings.

Variable	All (*n* = 231)	DCB group (*n* = 83)	DES group (*n* = 148)	*P* value
Pre-procedural findings				
Lesion length, mm	28.84 ± 5.19	29.14 ± 5.51	28.67 ± 4.98	0.508
Maximum plaque burden, %	84.20 ± 4.99	84.16 ± 5.09	84.22 ± 4.93	0.932
Calcification in lesion, *n*%	30 (13.0%)	9 (10.8%)	21 (14.2%)	0.601
Maximum arc of calcium,°	122.56 ± 21.00	121.30 ± 19.37	123.27 ± 21.82	0.494
Dissection, *n*%	17 (7.4%)	14 (16.9%)	3 (2.0%)	<0.001
Dissection extended into an MB, *n*%	2 (0.9%)	2 (2.4%)	0 (0.0%)	0.128
Reference minimum lumen area, mm^2^	3.59 ± 1.59	3.73 ± 1.72	3.52 ± 1.51	0.324
Reference maximum plaque burden, %	58.08 ± 3.38	58.30 ± 3.10	57.96 ± 3.53	0.463
Acute lumen gain, mm	1.81 ± 0.35	1.58 ± 0.36	1.94 ± 0.26	<0.001
Pre-procedural MB segment				
Distance from LAD ostium to MB, mm	34.64 ± 7.18	34.80 ± 6.61	34.55 ± 7.48	0.800
Total MB length, mm	12.27 ± 2.19	12.09 ± 2.39	12.38 ± 2.07	0.346
Maximum thickness of MB, mm	0.47 ± 0.08	0.47 ± 0.07	0.47 ± 0.08	0.749
Diastolic vessel area at max compression site, mm^2^	4.35 ± 0.60	4.27 ± 0.56	4.40 ± 0.62	0.108
Diastolic vessel restriction, %	18.93 ± 4.27	18.61 ± 4.73	19.11 ± 3.98	0.392
Minimum lumen area, mm^2^	2.31 ± 0.56	2.30 ± 0.67	2.32 ± 0.48	0.867
Plaque burden at minimum lumen area site, %	41.34 ± 4.16	41.30 ± 3.99	41.36 ± 4.25	0.923
Post-procedural findings				
MSA, mm^2^	5.43 ± 2.93	5.57 ± 2.25	5.35 ± 3.25	0.581
Stent expansion, %	70.31 ± 3.04	70.28 ± 3.10	70.33 ± 3.01	0.915
Rate of MSA in the MB, when stented, *n*%	115 (49.8%)	39 (47.0%)	76 (51.4%)	0.617

Continuous variables were expressed as mean ± SD. Categorical variables were expressed as number (percentage).

DES, drug-eluting stents; DCB, drug-coated balloons; MB, myocardial bridge; MSA, minimum stent area; LAD, left anterior descending artery; PCI, percutaneous coronary intervention;.

All MB segment parameters were measured pre-intervention using IVUS, prior to any balloon inflation or stent implantation.

### Angiographic findings at 12 months

At the 12-month follow-up, both groups showed similar minimum lumen diameter (2.60 ± 0.43 mm vs. 2.55 ± 0.37 mm, *p* = 0.313) and diameter stenosis (19.16% vs. 19.01%, *p* = 0.673). However, LLL was significantly lower in the DCB group (−0.04 ± 0.04 mm) compared to the DES group (0.18 ± 0.05 mm, *p* < 0.001). Restenosis occurred in 3.6% of DCB patients and 13.5% of DES patients (*p* = 0.029), while no cases of thromboembolism were reported in either group (Table 4).

**Table 4:** Angiographic findings at 12 months post-procedure.

Variable	All (*n* = 231)	DCB group (*n* = 83)	DES group (*n* = 148)	*P* value
Minimum lumen diameter, mm	2.57 ± 0.39	2.60 ± 0.43	2.55 ± 0.37	0.313
Diameter stenosis, %	19.06 ± 2.66	19.16 ± 2.33	19.01 ± 2.84	0.673
LLL, mm	0.10 ± 0.12	−0.04 ± 0.04	0.18 ± 0.05	<0.001
Restenosis, *n*%	23 (10.0%)	3 (3.6%)	20 (13.5%)	0.029
Thromboembolism, *n*%	0 (0.0%)	0 (0.0%)	0 (0.0%)	–
Stent fracture, *n*%	1 (0.4%)	0 (0.0%)	1 (0.7%)	1.000

Continuous variables were expressed as mean ± SD. Categorical variables were expressed as number (percentage).

DES, drug-eluting stents; DCB, drug-coated balloons; LLL, late lumen loss;.

### Clinical outcomes at 12 months

**Figure 2:**
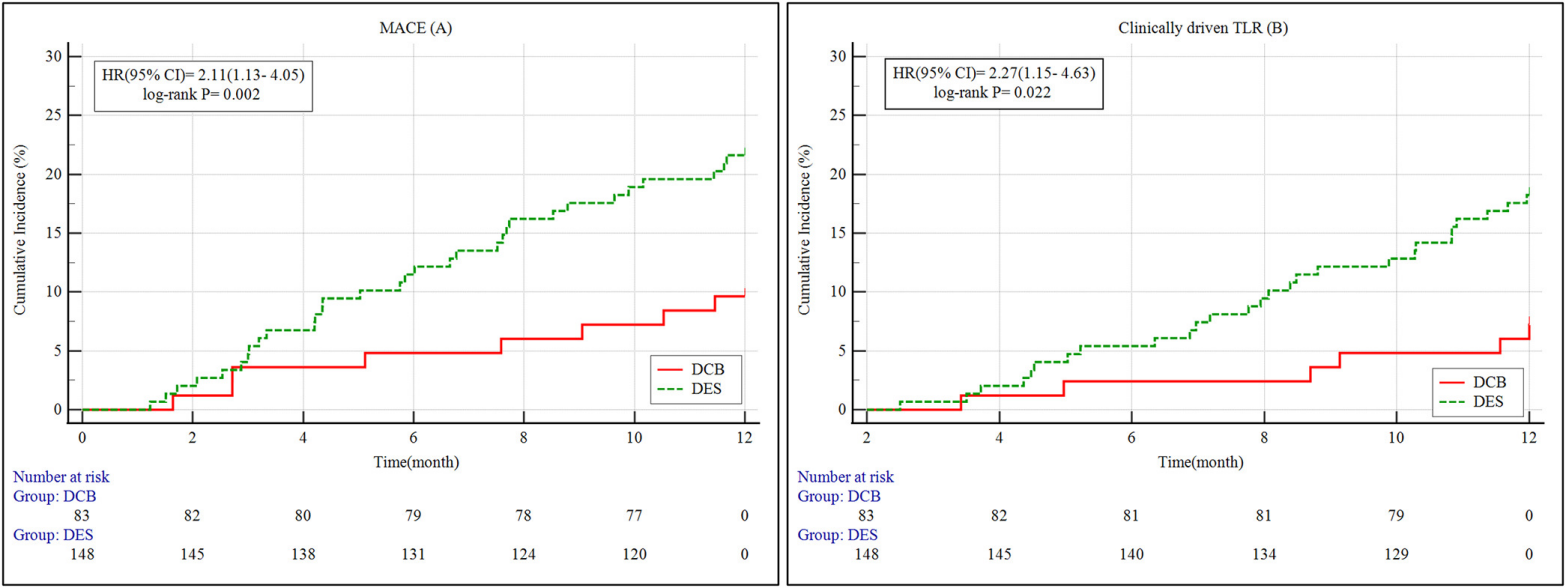
A + B: Kaplan–meier survival curves of MACE and clinically driven TLR for 12 months. MACE, major adverse cardiovascular events; 95% CI, 95% confidence intervals; HR, hazard ratio; DES, drug-eluting stents; DCB, drug-coated balloons; TLR, target lesion revascularization.

The incidence of MACE was significantly lower in the DCB group (9.6%) compared to the DES group (21.6%, *p* = 0.033). Clinically driven TLR was also reduced in the DCB group (7.2% vs. 18.2%, *p* = 0.035). No significant differences were found for cardiac death (0.0% vs. 0.7%, *p* = 1.000), target vessel MI (3.6% vs. 1.4%, *p* = 0.507), or rehospitalization due to recurrent angina (6.0% vs. 4.1%, *p* = 0.724) (Table 5 and Figure 2).

**Table 5:** 12 months clinical outcomes.

Variable	All (*n* = 231)	DCB group (*n* = 83)	DES group (*n* = 148)	*P* value
MACE, *n*%	40 (17.3%)	8 (9.6%)	32 (21.6%)	0.033
Cardiac death, *n*%	1 (0.4%)	0 (0.0%)	1 (0.7%)	1.000
Target vessel MI, *n*%	5 (2.2%)	3 (3.6%)	2 (1.4%)	0.507
Clinically driven TLR, *n*%	33 (14.3%)	6 (7.2%)	27 (18.2%)	0.035
Rehospitalization due to recurrent angina, *n*%	11 (4.8%)	5 (6.0%)	6 (4.1%)	0.724
Minor bleeding complications, *n*%	10 (4.3%)	2 (2.4%)	8 (5.4%)	0.461

Categorical variables were expressed as number (percentage).

DES, drug-eluting stents; DCB, drug-coated balloons; MACE, major adverse cardiovascular events; MI, myocardial infarction; TLR, target lesion revascularizatio.

### Predictors of MACE

Univariate Cox regression identified LLL (HR = 2.58, 95% CI: 1.49–4.46, *p* = 0.001), acute lumen gain (HR = 1.74, *p* = 0.021), dissection (HR = 1.91, *p* = 0.029), and cutting/scoring balloon use (HR = 1.69, *p* = 0.037) as significant predictors of MACE. In multivariate analysis, only LLL remained an independent predictor (HR = 2.43, 95% CI: 1.41–4.18, *p* = 0.002), indicating its strong prognostic relevance (Table 6).

**Table 6:** Univariate and multivariate cox regression analyses showing independent predictors of MACE.

Variables	Univariate analysis			Multivariate analysis		
	HR	95% CI	*P* value	HR	95% CI	*P* value
Age (per 10 years)	1.08	0.91–1.28	0.370	–	–	–
Male sex	1.12	0.67–1.87	0.650	–	–	–
Hypertension	0.92	0.53–1.58	0.750	–	–	–
Diabetes mellitus	1.25	0.68–2.28	0.470	–	–	–
LLL (mm)	2.58	1.49–4.46	0.001	2.43	1.41–4.18	0.002
Restenosis	1.45	0.70–3.00	0.320	–	–	–
Acute lumen gain (mm)	1.74	1.08–2.81	0.021	1.21	0.76–1.94	0.410
Dissection	1.91	1.07–3.42	0.029	1.36	0.72–2.56	0.340
Cutting/Scoring balloon use	1.69	1.03–2.79	0.037	1.22	0.72–2.05	0.460
Non-compliant balloon use	0.88	0.53–1.46	0.610	–	–	–
Semi-compliant balloon use	1.03	0.51–4.21	0.933			
DCB vs. DES group	1.30	0.60–3.19	0.415			
Balloon type	1.21	0.90–1.81	0.192			

LLL was analyzed only in patients who were MACE-free at the time of 12-month follow-up angiography. Balloon type was categorized into four types: semi-compliant balloons, non-compliant balloons, cutting balloons, and scoring balloons. These were treated as categorical variables in the multivariate analysis, with semi-compliant balloons serving as the reference group.

MACE, major adverse cardiovascular events; HR, hazard ratios; CI, confidence interval; LLL, late lumen loss.

## Discussion

**Figure 3:**
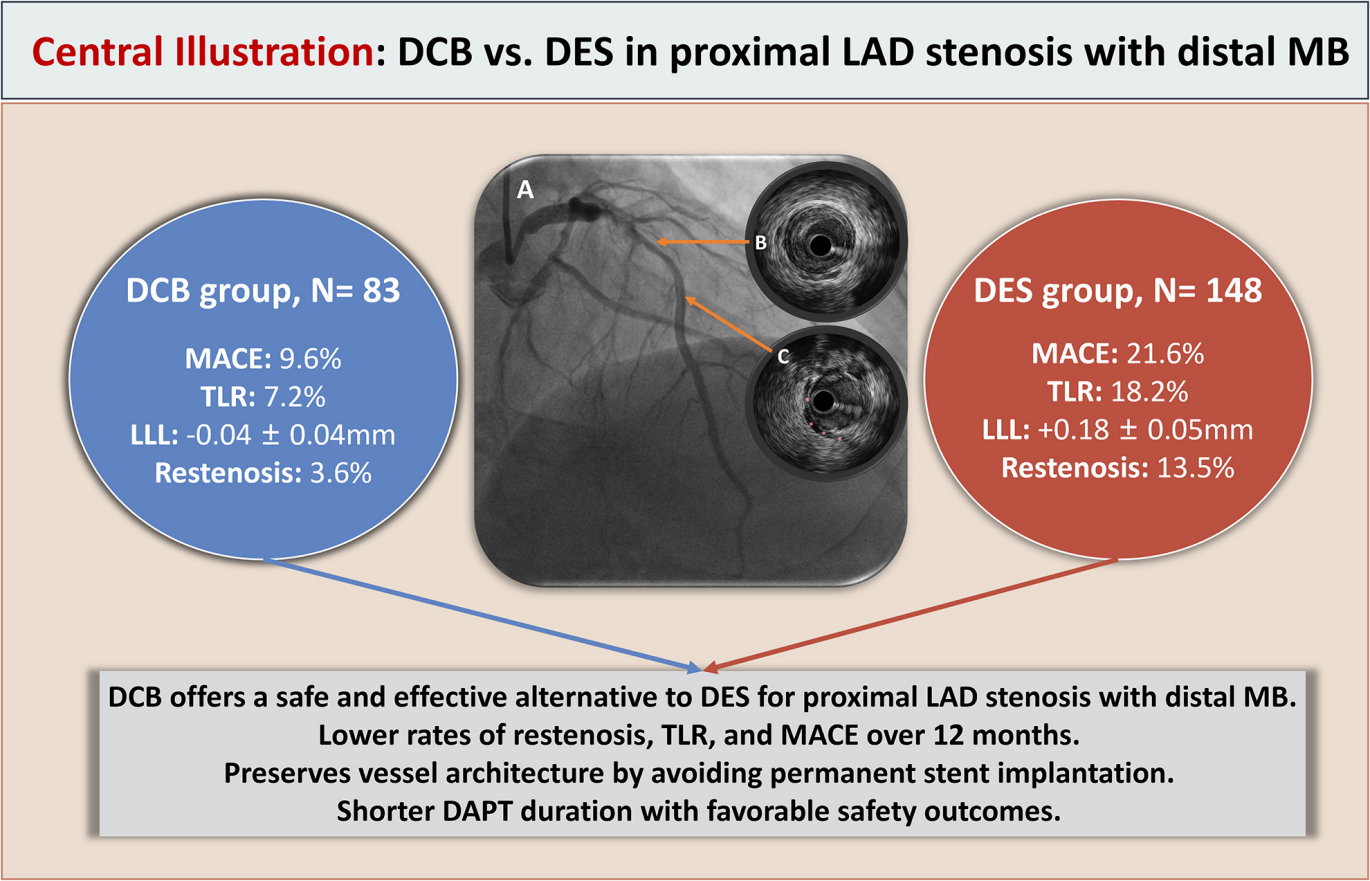
**Central illustration**. DCB vs. DES treatment for proximal LAD artery stenosis with distal MB. **(A)** Coronary angiography showing proximal LAD artery stenosis with a mid-segment MB. **(B)** IVUS image demonstrating significant plaque-induced stenosis in the proximal LAD segment. **(C)** IVUS image illustrating the myocardial bridge segment; the pink asterisk (*) indicates the overlying MB. DCB, indicates drug-coated balloon; DES, drug-eluting stent; LAD, left anterior descending artery; MB, myocardial bridge; MACE, major adverse cardiovascular events; TLR, target lesion revascularization; LLL, late lumen loss; IVUS, intravascular ultrasound; DAPT, dual antiplatelet therapy.

This study provides evidence that DCB represent a viable and effective revascularization modality for patients presenting with proximal LAD artery stenosis in the presence of a distal MB. Compared to DES, the use of DCBs was associated with a reduced incidence of target lesion restenosis and MACE over a 12-month follow-up period. Although the DCB group exhibited a relatively smaller acute lumen gain, this did not compromise midterm clinical outcomes. On the contrary, lesions treated with DCBs demonstrated superior vessel preservation, LLL, and lower rates of clinically driven TLR. The avoidance of a permanent metallic scaffold likely contributed to preserved vessel compliance and motion—features that may be particularly beneficial in anatomically dynamic segments such as those affected by MB. Importantly, multivariate analysis identified LLL as an independent predictor of MACE, underscoring the importance of maintaining sustained luminal patency in influencing long-term prognosis (Figure 3: Central Illustration).

Managing coronary stenosis with adjacent MB presents unique technical and physiological challenges for interventional cardiologists. Previous studies have reported elevated rates of stent-related complications in MB patients, especially when DES are deployed near or within the bridged segment. In the present cohort, individuals with proximal LAD lesions and distal MB treated using DCBs experienced lower MACE and TLR rates at 12 months compared to those receiving DES. Angiographic reassessment suggested that restenosis was the principal driver of TLR, and its incidence was significantly reduced in the DCB group. These results are in alignment with previous findings. For example, Zhang et al. (14) showed increased MACE rates following DES implantation in LAD stenosis patients with MB compared to those without. Similarly, Tsujita et al. (28) emphasized the negative impact of inadvertent stent extension into the bridged segment, which correlated with adverse outcomes. In contrast, the DCB strategy in our study may have mitigated these mechanical challenges by preserving native vessel architecture and avoiding metallic intrusion into MB zones. When compared to the study by Jeger et al. (17) evaluating DCBs in small vessel disease without MB involvement, our observed complication rates were marginally higher, likely reflecting the complex hemodynamic environment introduced by MB. This includes repetitive systolic compression and abnormal shear stress, which are known to contribute to endothelial dysfunction and recurrent ischemic episodes. Supporting this, Lee et al. (29) reported a nearly threefold increase in MACE incidence among MB patients treated with DES compared to non-MB counterparts. Although the incidence of post-procedural angina was comparable between groups, the DCB group demonstrated a significantly lower cumulative MACE rate. It is noteworthy that persistent anginal symptoms in this patient population may not be entirely attributable to fixed stenosis, but could also result from MB-induced coronary vasospasm—a dynamic pathology not effectively addressed through conventional stenting. Taken together, these findings suggest that although DCB therapy may be slightly less favorable in non-MB small vessel disease, it offers substantial clinical benefit in MB-associated anatomies. Specifically, DCBs may reduce ischemia-driven events and avoid the mechanical complications linked to DES implantation, making them a compelling alternative in this anatomically challenging subgroup. Recent studies have further reinforced the evolving role of DCBs in *de novo* lesions, including complex anatomies such as bifurcations, small vessels, and ostial disease. Evidence summarized in recent expert reviews indicates that DCBs, when applied with meticulous lesion preparation, offer favorable safety and efficacy outcomes across a range of complex lesion subsets. These emerging data support the broader application of DCB strategies beyond traditional in-stent restenosis or bailout scenarios and align with our findings, particularly in anatomically complex subsegments influenced by MB (30).

The PICCOLETO trial was the first randomized study to compare paclitaxel-coated balloons with DES in patients with small vessel CAD (31). Although the trial was pioneering in concept, it was prematurely terminated after enrolling approximately two-thirds of the intended sample size due to a significantly higher rate of restenosis in the DCB group, as well as a trend toward increased MACEs at 9 months. Subsequent analysis attributed the poor outcomes primarily to inadequate lesion preparation. Notably, only 25% of patients in the DCB arm underwent predilatation, compared with 86.2% in the DES arm. Moreover, no advanced lesion modification tools—such as cutting or scoring balloons—were utilized, which may have further compromised the effectiveness of drug delivery and procedural success.

In contrast to the PICCOLETO protocol, our study implemented a standardized, evidence-based approach to lesion preparation, guided by IVUS imaging and expert consensus recommendations (15, 16). All lesions received comprehensive pre-dilatation prior to DCB application, and a significantly higher proportion of cutting and scoring balloons were employed in the DCB group relative to the DES group. This strategy was associated with a notable reduction in both restenosis rates and MACEs, while minimizing the need for bailout stenting. Only one patient in the DCB cohort required rescue stent implantation due to a type C dissection. The remaining angiographic dissections were primarily type A or B and resolved spontaneously on follow-up angiography. Previous studies (32) have also suggested that non–flow-limiting dissections (i.e., type A to C) following optimal lesion preparation may be associated with favorable outcomes, including late lumen enlargement through positive vascular remodeling. These findings emphasize the critical role of individualized and thorough lesion preparation when employing DCB therapy, particularly in anatomically challenging settings such as distal MB-involved lesions.

During the 12-month follow-up, QCA revealed no significant difference in baseline MLD between the DCB and DES groups. Although the DES group achieved greater acute lumen gain immediately post-procedure, both groups demonstrated comparable MLD and percent diameter stenosis at follow-up. Importantly, the DCB group exhibited significantly smaller LLL, indicating a potential trend toward favorable vessel remodeling over time. This observation aligns with prior findings (33) and may reflect a physiological advantage of DCB therapy in certain lesion subsets. Several mechanisms may underlie the enhanced remodeling observed with DCB treatment (15). First, in vessels with large diameters, substantial plaque burden, or extensive calcific or fibrotic changes, challenges such as acute recoil or limited device deliverability are common. As DCBs lack the structural support of a stent, adequate lesion preparation becomes paramount. In our study, cutting and scoring balloons were more frequently used in the DCB group, consistent with expert guidelines. These specialized devices facilitate intimal disruption, improving drug absorption and reducing the risk of recoil compared to conventional balloon angioplasty (34). The mechanical scoring of plaque weakens medial resistance and allows for controlled expansion with fewer flow-compromising dissections. Over time, this may be further augmented by hemodynamic forces and improved endothelial healing, resulting in sustained luminal gain. Second, the absence of a permanent metallic implant with DCB therapy preserves native vascular architecture. Unlike DES, which leave a scaffold that may provoke chronic inflammation and stimulate neointimal proliferation, DCBs allow for natural vessel healing without ongoing foreign-body reaction. These factors collectively contribute to favorable late vessel remodeling and may compensate for the smaller acute gain observed with DCBs. This highlights the value of rigorous lesion preparation and supports the unique biological benefits of DCB therapy in appropriately selected coronary lesions (15).

A study conducted by Corballis et al. (35) demonstrated that the incidence of thromboembolic complications remained notably low following DCB angioplasty, even when DAPT was limited to a duration of only 1 month over a 6-month observation period. In alignment with these findings, patients in the DCB group of our study were treated with a short-term DAPT regimen of 1 month, while those in the DES group received standard 12-month DAPT in accordance with guideline-based protocols. Importantly, no cases of definite or probable stent thrombosis were reported during the follow-up period in either group. Additionally, no major bleeding events occurred, and the incidence of minor bleeding events remained low across both cohorts. These outcomes reinforce the safety of abbreviated DAPT protocols following DCB treatment and suggest potential advantages in carefully selected populations—particularly those at elevated risk for bleeding or with anticipated need for early non-cardiac surgical procedures. The favorable safety profile of DCBs may be attributable to their lack of a permanent intravascular scaffold and the absence of pro-thrombotic polymer coatings, which are commonly associated with DES. Furthermore, shorter DAPT durations may offer additional practical benefits, including improved patient adherence to pharmacotherapy, reduced anxiety related to hemorrhagic risk, and decreased healthcare expenditures. Collectively, these findings suggest that DCB-guided PCI represents a promising therapeutic alternative for patients in whom prolonged DAPT is either contraindicated or clinically undesirable.

## Limitations

This study has several limitations that should be acknowledged. First, the investigation was conducted at a single tertiary care center with a relatively modest sample size, which may limit the external validity and generalizability of the findings to wider clinical populations. Second, although IVUS was systematically applied in all cases, variations in image quality, interpretation, or operator technique over the 6-year inclusion period may have introduced analytical variability. This may have introduced heterogeneity in lesion characterization and procedural decision-making. Third, the follow-up period was restricted to 12 months, which, although adequate for assessing short-term safety and efficacy, is insufficient to capture late restenosis, stent-related complications, or progressive vascular remodeling. Future prospective studies with extended follow-up durations are warranted to evaluate long-term vessel behavior and clinical outcomes. Fourth, the absence of functional assessments—such as fractional flow reserve (FFR) or instantaneous wave-free ratio (iFR)—limits the ability to physiologically confirm ischemia, particularly in cases complicated by MB, where anatomical severity may not fully reflect true ischemic burden. This limitation could also have influenced treatment decision-making (e.g., DCB vs. DES) in borderline lesions, and should be considered when interpreting the therapeutic appropriateness. Fifth, this study was designed as a retrospective, non-randomized analysis, which inherently introduces a risk of selection bias. Although uniform inclusion criteria and imaging-guided protocols were applied, the allocation to DCB or DES groups was ultimately influenced by clinical judgment and operator preference, which may have affected baseline comparability. Although baseline characteristics were well-balanced between groups, propensity score matching analysis was not performed, and residual confounding cannot be fully excluded. Furthermore, the recruitment period spanned six years (2017–2023), during which changes in clinical practice, device technology, and operator experience may have occurred. This temporal heterogeneity introduces the possibility of time-related bias, which may confound the interpretation of outcome differences between groups. Sixth, the study did not incorporate a formal cost-effectiveness analysis. Given the potential application of DCB in patient populations at high bleeding risk or requiring early surgical intervention, economic considerations are essential to support broader clinical adoption of this therapeutic strategy. Finally, the imbalance in lesion preparation techniques (e.g., higher use of cutting or scoring balloons in the DCB group) may have influenced angiographic and clinical outcomes. These devices are not neutral tools—they can enhance acute lumen gain and drug uptake, potentially introducing performance bias favoring the DCB group. Although statistical adjustment was applied, residual confounding cannot be entirely excluded. Similarly, the duration of DAPT differed substantially between groups (1 month in the DCB group vs. 12 months in the DES group), which may have influenced safety outcomes such as MACE or bleeding events. As DAPT duration was predetermined by treatment strategy, it could not be independently adjusted for in multivariable modeling, and should be interpreted as a potential source of confounding.

## Conclusion

In conclusion, DCB angioplasty appears to offer a safe and effective revascularization strategy for patients with proximal LAD artery stenosis coexisting with distal MB. Despite yielding a smaller acute lumen gain compared to DES, DCB treatment was associated with lower incidences of restenosis, TLR, and MACEs over a 12-month follow-up period. The absence of a permanent intravascular scaffold likely contributes to the preservation of native vessel architecture and may reduce the risk of mechanical complications, particularly in segments exposed to dynamic systolic compression due to MB. Furthermore, the implementation of a short-duration DAPT regimen in the DCB group was associated with favorable safety outcomes, including low rates of thrombotic and bleeding events—offering potential advantages for patients with elevated bleeding risk or those requiring early surgical intervention. When combined with meticulous lesion preparation and intravascular imaging guidance, DCB therapy may serve as a compelling alternative to DES in anatomically complex coronary lesions, especially in the context of MB, where preserving vessel physiology is of critical importance.

## Data Availability

The raw data supporting the conclusions of this article will be made available by the authors, without undue reservation.
